# Ocean circulation contributes to genetic connectivity of limpet populations at deep‐sea hydrothermal vents in a back‐arc basin

**DOI:** 10.1111/eva.13727

**Published:** 2024-06-17

**Authors:** Yuichi Nakajima, Masako Nakamura, Hiromi Kayama Watanabe, Jun‐ichiro Ishibashi, Hiroyuki Yamamoto, Satoshi Mitarai

**Affiliations:** ^1^ Marine Biophysics Unit Okinawa Institute of Science and Technology Graduate University Okinawa Japan; ^2^ Center for Climate Change Adaptation National Institute for Environmental Studies Tsukuba Ibaraki Japan; ^3^ School of Marine Science and Technology Tokai University Shizuoka Japan; ^4^ Institute for Extra‐Cutting‐Edge Science and Technology Avant‐Garde Research (X‐Star) Japan Agency for Marine‐Earth Science and Technology (JAMSTEC) Yokosuka Kanagawa Japan; ^5^ Kobe Ocean‐Bottom Exploration Center Kobe University Kobe Hyogo Japan; ^6^ Marine Biodiversity and Environmental Assessment Research Center (BioEnv), Research Institute for Global Change (RIGC) Japan Agency for Marine‐Earth Science and Technology (JAMSTEC) Yokosuka Kanagawa Japan

**Keywords:** genetic structure, microsatellites, migration, ocean circulation model, Okinawa Trough

## Abstract

For endemic benthos inhabiting hydrothermal vent fields, larval recruitment is critical for population maintenance and colonization via migration among separated sites. The vent‐endemic limpet, *Lepetodrilus nux*, is abundant at deep‐sea hydrothermal vents in the Okinawa Trough, a back‐arc basin in the northwestern Pacific; nonetheless, it is endangered due to deep‐sea mining. This species is associated with many other vent species and is an important successor in these vent ecosystems. However, limpet genetic diversity and connectivity among local populations have not yet been examined. We conducted a population genetics study of *L. nux* at five hydrothermal vent fields (maximum geographic distance, ~545 km; depths ~700 m to ~1650 m) using 14 polymorphic microsatellite loci previously developed. Genetic diversity has been maintained among these populations. Meanwhile, fine population genetic structure was detected between distant populations, even within this back‐arc basin, reflecting geographic distances between vent fields. There was a significant, positive correlation between genetic differentiation and geographic distance, but no correlation with depth. Contrary to dispersal patterns predicted by an ocean circulation model, genetic migration is not necessarily unidirectional, based on relative migration rates. While ocean circulation contributes to dispersal of *L. nux* among vent fields in the Okinawa Trough, genetic connectivity may be maintained by complex, bidirectional dispersal processes over multiple generations.

## 1 INTRODUCTION

Many invertebrate species endemic to deep‐sea hydrothermal vents have developed symbiotic relationships with chemoautotrophic bacteria (reviewed in Tunnicliffe, 1992; Van Dover, 2000). As hydrothermal vent fields are now facing disturbances caused by polymetallic sulfide mining, more than a hundred vent‐endemic species are listed as endangered in the International Union for Conservation of Nature Red List of Threatened Species (Sigwart et al., 2019; Thomas et al., 2021, 2022). Genetic diversity and connectivity among populations are generally required to maintain flourishing vent ecosystems, which constitute an element of sustainable and resilient oceans. Adults of many hydrothermal vent species are benthic; therefore, dispersal that connects the populations and maintains genetic diversity of metapopulation is mediated by pelagic larvae or mobile juveniles (Vrijenhoek, 2010). For benthos endemic to hydrothermal vent environments, larval recruitment is critical for population maintenance and colonization via migration among populations, as vent fields are patchy on the seafloor (Adams et al., 2012). Long‐distance planktonic larval dispersal contributes to genetic diversity and connectivity among vent‐field populations, and may help to mitigate degradation caused by disturbances. Most larvae die due to predation and lethal stresses during planktonic dispersal. Surviving larvae that manage to reach other hydrothermal vent fields, settle and grow. Larvae mature and reproduce to leave a genetically traceable footprint in the local population. Long‐distance larval dispersal likely occurs stochastically in stepping‐stone fashion over many generations (Geiger & Thacker, 2005; Vrijenhoek, 1997). Therefore, recruitment among hydrothermal vent fields appears to have contributed to expansion of species distributions at a historical timescale, and maintenance of metapopulations at an ecological timescale. Mitarai et al. (2016) predicted the probability of larval dispersal among hydrothermal vents using an ocean circulation model, and suggested possible connections between hydrothermal vent fields.

Lecithotrophic larvae, which are dependent on nutrition derived from egg yolk, appear to have shorter planktonic larval durations and generally more limited dispersal potential than planktotrophic larvae, which are able to feed while dispersing (Lutz et al., 1984; Lutz & Kennish, 1993; Vrijenhoek, 2010). Nonetheless, larval nutrition type alone may not determine actual larval dispersal distance, as larval duration in lecithotrophic species may be prolonged by production of large eggs or arrested development (Vrijenhoek, 2010). Moreover, empirical assumptions about larval dispersal may not apply to deep‐sea species due to colder water temperatures. Lower temperatures and slower metabolism can prolong larval duration, although they also increase mortality due to increased predation and severe environmental conditions (O'Connor et al., 2007). Most hydrothermal vent gastropods appear to have lecithotrophic larvae (Tyler et al., 2008). It is likely that *Lepetodrilus* is no exception (Craddock et al., 1997; Johnson et al., 2006, 2008; Plouviez et al., 2009; Vrijenhoek, 2010), though the egg size is suggestive of planktotrophic larval development (Tyler et al., 2008). Nevertheless, a low rate of successful larval dispersal appears sufficient to maintain connectivity among sites (Lowe & Allendorf, 2010).

Population genetic studies using genetic markers are essential for estimation of population connectivity among geographically isolated sites. Since, genetic connectivity is influenced by larval dispersal because benthic animals disperse during the larval stage, vent gastropod species are genetically differentiated by geographic distance between regions, that is, horizontal distance (*Ifremeria nautilei*; Tran Lu et al., 2022) or between depths, that is, vertical distance (*Lepetodrilus concentricus*, formerly *Lepetodrilus* sp.; Roterman et al., 2016). However, there is little genetic differentiation within regions in *Lepetodrilus nux* (Nakamura et al., 2014). In the case of *Shinkailepas*, a planktotrophic limpet, there is no significant differentiation between regions separated by 1000 km (Yahagi et al., 2017, 2019, 2020). In vent gastropods, genetic connectivity has been studied using one to several genetic loci from mitochondrial and/or nuclear gene(s) (Breusing et al., 2023; Nakamura et al., 2014; Thaler et al., 2011; Yahagi et al., 2017, 2019, 2020). Meanwhile, high‐resolution genetic markers for vent species are expected to detect fine genetic connectivity, and some studies assessed connectivity using microsatellites or single‐nucleotide polymorphisms (SNPs) (Roterman et al., 2016; Tran Lu et al., 2022). Studies of regional genetic connectivity based on multilocus genotypes between hydrothermal vents are scarce, due to the difficulty of collecting samples. Nonetheless, genetic markers have helped to illuminate population structure and dynamics of larval dispersal in population maintenance of deep‐sea hydrothermal vent species (Roterman et al., 2016; Thaler et al., 2011). In addition, next‐generation sequencing has facilitated development of polymorphic microsatellite markers for population genetic analysis.

The Okinawa Trough is a back‐arc basin along the Ryukyu Archipelago in the northwestern Pacific Ocean. This trough is approximately 1000 km in length and more than 10 hydrothermal vent fields have been discovered in it to date (Fujikura et al., 2008; Miyazaki et al., 2017; Nakamura et al., 2015; Watanabe et al., 2010). However, some of these hydrothermal vent fields have been disturbed by scientific drilling or mineral exploration (Kawagucci et al., 2013). Populations of hydrothermal, vent‐endemic lepetodrilid limpets, *Lepetodrilus nux*, maintain considerable genetic diversity and are well‐mixed among vent fields in the Okinawa Trough, as illustrated using the mitochondrial COI gene (Nakamura et al., 2014). In this study, we documented genetic diversity and connectivity of *L. nux* using 14 polymorphic microsatellite loci developed using next‐generation sequencing (Nakajima et al., 2018). Now it is possible to gather more detailed genetic diversity and connectivity data using microsatellites, to better understand mechanisms of population maintenance in *L. nux*. *Lepetodrilus nux* is an endangered species (Molloy et al., 2020), but widely distributed at hydrothermal vents in the Okinawa Trough (Nakamura et al., 2014; Okutani et al., 1993; Sasaki et al., 2003). The genus *Lepetodrilus* is one of most diversified gastropod groups at deep‐sea hydrothermal vents (Desbruyères et al., 2006), and some *Lepetodrilus* species also colonize cold seeps, wood falls, and whale carcasses (Johnson et al., 2008). To consider the timescale for connectivity, we compared genetic connectivity to potential larval dispersal from the ocean circulation model (Mitarai et al., 2016). This model simulated dispersal times for hydrothermal vent sites using 55 years of data. Therefore, the model is appropriate for quantitative comparisons of population genetic connectivity and current ocean circulation patterns.

## 2 MATERIALS AND METHODS

### 2.1 Collection of samples and scoring of microsatellite genotypes

Specimens of *L. nux* were collected at five hydrothermal vent fields in the Okinawa Trough (maximum geographic distance: ~545 km, depth range: ~700 m to ~1650 m) (Table 1), using the remotely operated vehicle, Hyper‐Dolphin, during cruises NT11‐20 (September 29, 2011–October 12, 2011) and NT13‐22 (November 7–19, 2013) of the R/V Natsushima, conducted by the Japan Agency for Marine‐Earth Science and Technology. All specimens were preserved in 99.5% ethanol and then transferred to an onshore laboratory. Genomic DNA was extracted using a DNeasy Blood & Tissue Kit (Qiagen). We used 14 microsatellite loci from our specimens with efficient multiplex PCR amplification. These loci do not deviate from Hardy–Weinberg equilibrium (HWE) (Nakajima et al., 2018). The reaction mixture (5 μL) contained template DNA (<100 ng), 2× Type‐it Microsatellite PCR Master Mix (Qiagen), and three primers for each locus: a non‐tailed forward primer (0.1 μM), a reverse primer with a sequence tail (0.1 μM), and a U19, M13R, T7, or SP6 primer (0.1 μM) fluorescently labeled with FAM, VIC, NED, or PET, respectively (Schuelke, 2000). Combinations of loci and sequences of primers are described in Table S1. Amplification of all microsatellite loci was carried out under the following conditions: 95°C for 5 min; followed by 40 cycles at 95°C for 30 s, 55°C for 90 s, and 72°C for 30 s; and a final extension at 60°C for 30 min. Lengths of amplified PCR fragments were analyzed using internal size standards (GeneScan 600 LIZ, Thermo Fisher Scientific) on an automated, capillary‐based, DNA sequencer (ABI 3130xl Genetic Analyzer, Thermo Fisher Scientific) and Geneious ver. 9.0.4 (Biomatters Ltd.).

**TABLE 1:** Location information and genetic indices for *Lepetodrilus nux* at each sampled vent field in the Okinawa Trough.

Location	Code	Latitude (N)	Longitude (E)	Depth (m)	*N*	*A*_R_	*H*_O_	*H*_E_	*F*_IS_	IAM	TPM	*p*_ID_
Minami Ensei Knoll	MEK	28°23.47′	127°38.39′	~700	38	9.28	0.695	0.731	0.040	0.428	0.999	1.5 × 10^−15^
Iheya North Field	INF	27°47.41′	126°54.04′	~1058	25	9.50	0.751	0.735	−0.022	0.108	0.955	5.0 × 10^−16^
Izena Hole	IZH	27°14.81′	127°04.08′	~1617	39	8.94	0.674	0.759	0.117	0.015	0.837	1.5 × 10^−16^
Irabu Knoll	IRK	25°13.75′	124°52.20′	~1650	25	9.93	0.729	0.772	0.054	0.045	0.892	3.2 × 10^−17^
Hatoma Knoll	HTK	24°51.47′	123°50.50′	~1477	31	9.43	0.749	0.778	0.034	0.025	0.596	2.3 × 10^−17^

*Note*: *N* is the number of samples analyzed. *A*_R_ is allelic richness, the standardized index of genetic diversity. *H*_O_ and *H*_E_ are the mean observed and expected heterozygosities, respectively. *F*_IS_ is the fixation index (inbreeding coefficient), deviation from HWE. IAM and TPM are significant values of the infinite allele model and two‐phase model in the analysis using BOTTLENECK, respectively. *p*_ID_ is the probability of identify for each vent field.

### 2.2 Documenting genetic diversity

Numbers of alleles, values of observed and expected heterozygosity (*H*_O_ and *H*_E_, respectively), and a deviation index (*F*_IS_) from HWE were also evaluated with GenAlEx ver. 6.501 (Peakall & Smouse, 2006). For estimation of genetic diversity, we calculated allelic richness at each vent field, normalized for differences of sample size using FSTAT ver. 2.9.3.2 (Goudet, 1995). The expected *H*_E_ value was used as an index of genetic diversity. The probability of recent genetic bottlenecks was examined using BOTTLENECK ver. 1.2.02 (Piry et al., 1999) based on assumptions for both the infinite allele model (IAM) and the two‐phase model (TPM; 30% IAM and 70% stepwise mutation model). For this analysis, we adopted Wilcoxon's signed‐rank test with 1000 replications. The probability of identify (*p*_ID_) was also calculated with these 14 microsatellite loci, using GenAlEx to estimate the resolution of individual identification.

### 2.3 Genetic differentiation and population isolation

Genetic differentiation was estimated using hierarchical analysis of molecular variance (AMOVA) (Excoffier et al., 1992). Genetic differentiation indices between vent fields, pairwise *F*_ST_, and $G_{\mathrm{ST}}^{\prime \prime }$, were calculated using GenAlEx. The significance of each *F*_ST_ value was tested with 999 permutations. Furthermore, the *G*‐statistic method was used to calculate pairwise $G_{\mathrm{ST}}^{\prime \prime }$, which is effective for estimating genetic differentiation with a small number of populations (Meirmans & Hedrick, 2011), and the significance of each value was tested with 999 permutations. Isolation‐by‐distance was examined using the Mantel test in GenAlEx to determine whether the correlation between pairwise *F*_ST_/(1 – *F*_ST_) and $G_{\mathrm{ST}}^{\prime \prime }$/(1 – $G_{\mathrm{ST}}^{\prime \prime }$) values and direct geographic distances (km) between vent fields was significant. Isolation‐by‐depth was also analyzed by the same method. Furthermore, the isolation‐by‐distance pattern was compared to patterns of other vent species, including vent‐related species inhabiting both vent and seep sites, in the Okinawa Trough based on data published previously. Isolation‐by‐distance in published data was calculated from the genetic differentiation based on pairwise *F*_ST_ or Φ_ST_, and geographic distance was calculated from coordinates of vent sites. Published data are derived from analyses of the abyssochrysoid gastropod, *Provanna subglabra* (Ogura et al., 2018), the bathymodioline mussel, *Gigantidas platifrons* (formerly *Bathymodiolus platifrons*) (Xu et al., 2018), the patellogastropod limpet, *Bathyacmaea nipponica* (Xu et al., 2021), and the galatheoid squat lobster, *Shinkaia crosnieri* (Xu et al., 2024). These studies analyzed vent species at four or more sites in the Okinawa Trough and nearby regions. Isolation‐by‐distance patterns were divided into cases of vent‐only populations and mixed vent and seep populations because *L. nux* and *P. subglabra* are both considered vent‐endemic, whereas *G. platifrons*, *B. nipponica*, and *S. crosnieri* include both seep and vent populations.

### 2.4 Genetic clustering approaches

Population genetic structure was inferred using STRUCTURE ver. 2.3.4 (Pritchard et al., 2000). STRUCTURE analysis implements a Bayesian clustering algorithm to assign genotypes to clusters that minimize HWE and linkage disequilibrium. Ten replicate runs were conducted for each *K* between 1 and 7 with location prior information using the admixture model and assuming correlated allele frequencies (Falush et al., 2003). Each run consisted of 1,000,000 Markov chain Monte Carlo (MCMC) replications after burn‐in with 100,000 iterations. Optimal *K* was determined using the method of Evanno et al. (2005), as implemented in STRUCTURE HARVESTER (Earl & vonHoldt, 2012). Run data were merged with CLUMPAK (Kopelman et al., 2015). Discriminant Analysis of Principal Components (DAPC) was conducted in R ver. 3.4.4 (R Core Team, 2018) using the package, adegenet ver. 1.3‐9.2 (Jombart et al., 2010) to represent genetic patterns of each individual. This clustering method does not assume a particular model; therefore, it is free of assumptions about HWE and linkage disequilibrium. Clusters were predefined for each site and different distances among clusters were demonstrated in a scatterplot of individuals.

### 2.5 Directional relative migration rates between vent fields

We employed the divMigrate function (Sundqvist et al., 2016) in diveRsity ver. 1.9.90 (Keenan et al., 2013) in R to estimate relative migration rates between vent fields, with the classical measurement method of genetic differentiation using allele frequency data based on the infinite island model. Statistical methods used Jost's *D* (Jost, 2008). The maximum migration rate between sites is normalized as 1, and other pairwise sites are reported as relative migration rates (<1). An asymmetrical test to estimate unidirectional migration was conducted and 95% confidence intervals were calculated from 15,000 bootstrap samples. In addition, we tested the significance of the directionality difference for both horizontal (geographic distance) and vertical migration (difference of depth), using values of relative migration rates.

### 2.6 Estimation of potential long‐distance dispersal based on an ocean circulation model

Estimation of successful dispersal among five vent fields was based on the ocean circulation model from the Regional Ocean Modeling System (ROMS) (Mitarai et al., 2016), which covers the five vent fields in this study. Planktonic larval duration (PLD) was set to the water temperature for each depth in western Pacific vent fields, estimated by Mitarai et al. (2016) following the previous mean PLD data of 69 marine species, including planktotrophic and lecithotrophic larval species (O'Connor et al., 2007). This is because larval metabolism can be accelerated by higher water temperature, so larvae have shorter PLDs closer to the surface. For example, larval dispersal periods of six species of lecithotrophic mollusks were estimated as 15.4 days at 10°C and 8.6 days at 20°C. In contrast, those of planktotrophic mollusks were estimated for 10 species at 50.2 days and 27.3 days, respectively (O'Connor et al., 2007) (Figure S2). The ocean circulation model shows the number of successful dispersal events of passive larvae in 100 million independent dispersal events from each vent site (Mitarai et al., 2016). We calculated the probability based on the number of successful dispersal events that can connect a focal population to its natal population per 100 million dispersal events. The probability was assessed per generation and per two successive generations, to simplify connectivity among vent fields. The probability of two successive generations was assumed to represent stepping‐stone connectivity among vent fields. The probability was calculated for depths of 100, 300, 500, 700, and 1000 m. The probability of recruitment at MEK was not considered at 1000 m, because the vent occurs at ~700 m (Table 1). Furthermore, the probability of dispersal was estimated in both directions, from northeast to southeast and from southeast to northeast, as relative migration rates.

## 3 RESULTS

### 3.1 Genotyping of *Lepetodrilus nux*

Using 14 microsatellite loci (Table S1), we scored multilocus microsatellite genotypes of 167 individuals collected from five hydrothermal vent fields in the Okinawa Trough (Table 1). Complete multilocus genotypes were obtained for 158 individuals for use in subsequent analyses. The probability of identity for each vent field ranged from 2.3 × 10^−17^ to 1.5 × 10^−15^. There were no replicated multilocus genotypes; therefore, all individuals in our sample possess unique multilocus genotypes, confirming the utility of these 14 loci.

### 3.2 Genetic diversity and bottlenecks in populations

Mean allelic richness (*A*_R_), normalized for differences in sample size across 14 loci per vent field, ranged from 8.94 to 9.93 (Table 1). Sixty private alleles (*P*_A_) were detected in 14 loci at five vent fields (Table S2). Mean observed and expected heterozygosities (*H*_O_ and *H*_E_) ranged from 0.674 to 0.751 and from 0.731 to 0.778 (Table 1), respectively. The mean deviation index (*F*_IS_) from HWE ranged from −0.022 to 0.117 (Table S2). Although three vent fields (IZH, IRK, and HTK) appear to have experienced a recent bottleneck under the infinite allele model (IAM) (*p* < 0.05), no populations displayed such a bottleneck under the two‐phase model (TPM) (*p* < 0.05) (Table 1).

### 3.3 Genetic differentiation among hydrothermal vent fields

**FIGURE 1:**
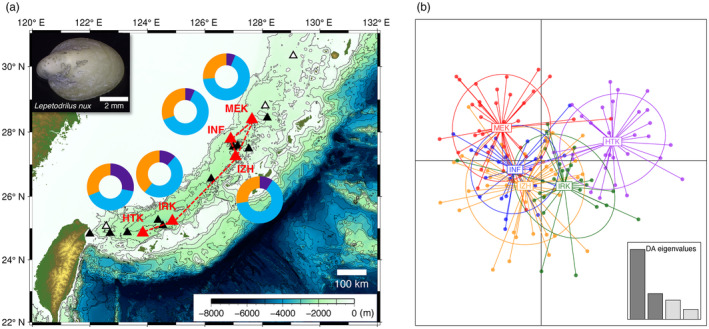
(a) Genetic clusters among five vent fields based upon the *Q*‐matrix, estimated by STRUCTURE analysis. The number of clusters are three (*K* = 3), and the optimal *K* was determined using the ∆*K* method of Evanno et al. (2005) (Figure S1a). Colors of the pie charts show each genetic cluster. Bar plots for each individual are shown in Figure S1b. Arrows show non‐significant differentiation in both *F*_ST_ and $G_{\mathrm{ST}}^{\prime \prime }$ between vent fields. Significance levels and pairwise *F*_ST_ and $G_{\mathrm{ST}}^{\prime \prime }$ values are shown in Table S3. Contour lines indicate sea depths at intervals of 500 m. (b) Discriminant analysis of principal components (DAPC) shows scatterplots with prior site information. Each plot explains multilocus genotypes analyzed using 14 microsatellites.

**FIGURE 2:**
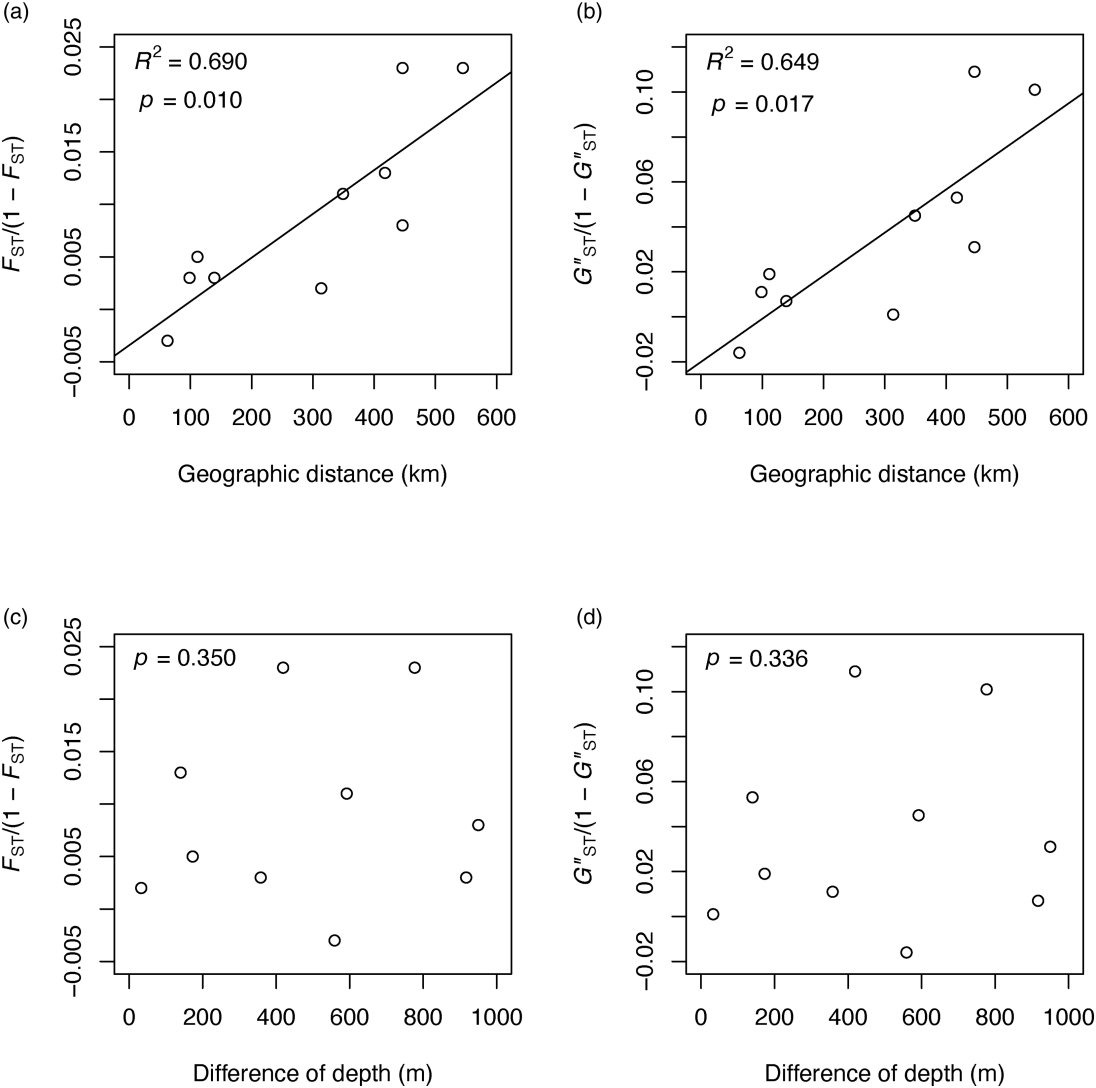
Isolation‐by‐distance (a, b) and isolation‐by‐depth (c, d) result from the *F*_ST_/(1 – *F*_ST_) (a, c) and $G_{\mathrm{ST}}^{\prime \prime }$/(1 – $G_{\mathrm{ST}}^{\prime \prime }$) (b, d) values of *Lepetodrilus nux* in the Okinawa Trough. Pairwise *F*_ST_ and $G_{\mathrm{ST}}^{\prime \prime }$ values are shown in Table S3.

Hierarchical AMOVA indicated significant genetic differentiation among vent fields (*F*_ST_ = 0.009, *p* = 0.001), and pairwise *F*_ST_ and $G_{\mathrm{ST}}^{\prime \prime }$ values ranged from −0.003 to 0.023 and from −0.016 to 0.098, respectively (Table S3). Significant genetic differentiation of both *F*_ST_ and $G_{\mathrm{ST}}^{\prime \prime }$ was confirmed between distant locations, whereas the closest hydrothermal vents did not show significant differentiation (Figure 1a). Supporting the results of *F*_ST_ and $G_{\mathrm{ST}}^{\prime \prime }$, STRUCTURE analysis with the LOCPRIOR model showed differences in the Q‐matrix among sites, especially those most separated. The Evanno method indicated that the most probable number of genetic populations was three, as the highest indicated Δ*K* was *K* = 3 (Figure S1a). However, genetic structure is not strong because each cluster is largely shared between vent fields (Figure 1a and Figure S1b). DAPC also documented differences in cluster composition between vent fields. In that analysis, the first and second discriminant functions explained 51.56% and 18.95% of the variance, respectively. Clusters, IRK and HTK, are especially isolated from the other three overlapping population clusters (Figure 1b). Those three clusters are closest to the IRK cluster, and the IRK cluster is most similar to the HTK cluster. There is no obvious differentiation in relation to depth (from ~700 m to ~1650 m) in this species. Isolation‐by‐distance was significant (*p* = 0.010 in *F*_ST_, *p* = 0.017 in $G_{\mathrm{ST}}^{\prime \prime }$) (Figure 2a,b), but isolation‐by‐depth was not confirmed (*p* = 0.350 in *F*_ST_, *p* = 0.336 in $G_{\mathrm{ST}}^{\prime \prime }$) (Figure 2c,d).

### 3.4 Directional relative migration rates

**FIGURE 3:**
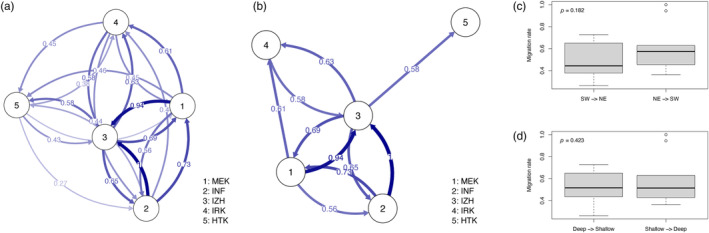
(a) Relative migration rates among populations of *Lepetodrilus nux* at five vent fields in the Okinawa Trough estimated with divMigrate. The most plausible migration rate was assumed as 1 (from INF to IZH), and relative migration patterns were output for other vent fields in pairwise fashion. An asymmetrical test revealed that all unidirectional migration patterns were non‐significant at a 95% threshold. (b) Relative migration rates between vent fields were over 0.5. Relative migration rates in each direction by geographic distance (c), and by depth (d), respectively. Migration from IZH to INF was counted as southwest to northeast in this analysis, and vice versa.

divMigrate analysis revealed gene flow among vent fields. A pattern of isolation‐by‐distance was detected based upon *F*_ST_ and $G_{\mathrm{ST}}^{\prime \prime }$ values. The highest migration rate occurred between INF and IZH, and the lowest rate was from HTK to MEK. These results also reflect geographic distance (Figure 3a). All migration rates among MEK, INF, and IZH exceeded 0.5, regardless of direction (Figure 3b). In addition, the migration rate was higher from northeast to southwest than the rate from southwest to northeast (Figure 3c); however, the two migration rates are not significantly different. Furthermore, mutual migration patterns between deep and shallow vent fields were detected (Figure 3d).

### 3.5 Comparison of isolation‐by‐distance with other species

For vent‐only populations, we detected isolation‐by‐distance in *G. platifrons*, analyzed by genome‐wide SNPs (Figure S3a) regardless of planktotrophic nutrient larval type and with longer PLD. In contrast, the other three species (*P. subglabra*, *B. nipponica*, and *S. crosnieri*) and *G. platifrons* analyzed by mtDNA did not show isolation‐by‐distance (Figure S3a). However, *B. nipponica* showed relatively high values of *F*_ST_ (ranging from 0.0075 to 0.3431), whereas *S. crosnieri* showed low *F*_ST_ values ranging from −0.0187 to −0.0040.

For both vent and seep populations, we did not detect significant isolation‐by‐distance in any of the four vent‐related species. In terms of SNPs, *G. platifrons* also did not show isolation‐by‐distance due to inclusion of seep populations, including the Jiaolong Ridge population in the South China Sea, which is relatively near the Okinawa Trough (Figure S3b). *Shinkaia crosnieri* did not exhibit isolation‐by‐distance, but it displayed large genetic differentiation between vent and seep populations with higher *F*_ST_ values ranging from −0.0187 to 0.2301 (Figure S3b).

### 3.6 Probability of dispersal based on the ocean circulation model

**FIGURE 4:**
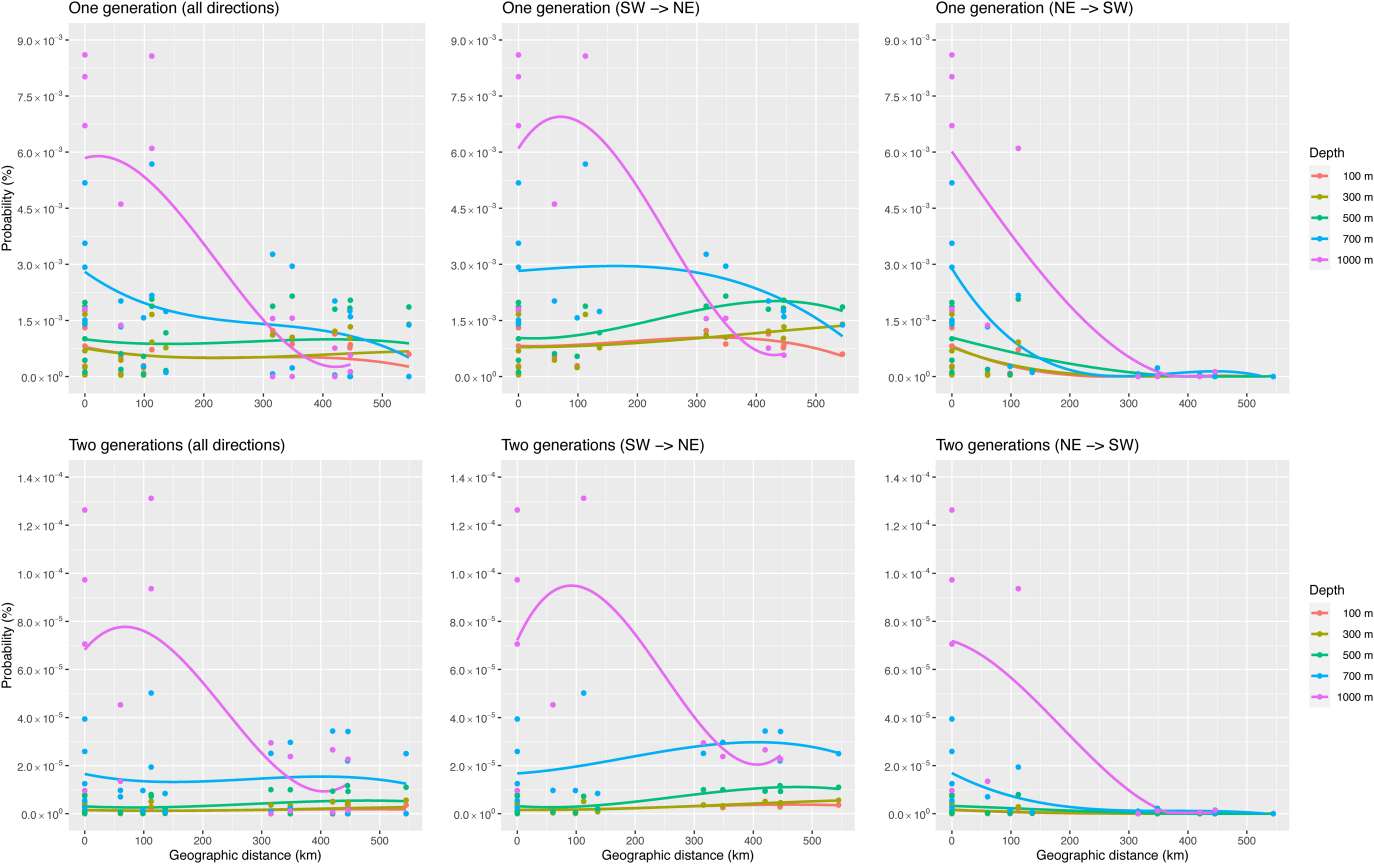
The probability of dispersal between hydrothermal vent fields estimated using the ocean circulation model (Mitarai et al., 2016). The probability of larval transport between hydrothermal vent populations per generation and per two successive generations at each depth (100, 300, 500, 700, and 1000 m). Data related to MEK (~700 m depth) could not be determined at 1000 m. Furthermore, dispersal probability is estimated in each direction. The direction between IZH and INF was considered a case of relative migration rates. Plot at 0 km of geographic distance means probability of self‐recruitment or return at each vent field. The polynomial regression curve was produced using ggplot2 package in R.

The probability of successful dispersal is relatively higher in deep‐water (Figure 4). Maximum probabilities are 0.00568% at 700 m and 0.00860% at 1000 m per generation, assuming 100 million independent events. In addition, maximal probabilities of two‐generation dispersal were also higher in deeper water, 0.00502% at 700 m and 0.01312% at 1000 m. However, the probability of two‐generation dispersal in shallow water showed a lower value (0.00051%) at 100 m depth. Northward dispersal was relatively higher regardless of depth, both per generation and per two successive generations. Meanwhile, the relative migration rate using a genetic approach shows non‐significant bidirectional migration patterns (*p* = 0.182) (Figure 3c).

## 4 DISCUSSION

*Lepetodrilus nux* populations in the Okinawa Trough show that there is no recent bottleneck effect at any vent field in this study. Genetic differentiation and clustering based on microsatellites reflect geographic distance, in contrast to results using the mitochondrial COI gene. Nevertheless, these genetic data appear to support a low level of genetic differentiation of *L. nux* in the Okinawa Trough. Genetic connectivity has been maintained, even at MEK, which is relatively shallow, compared to the other four vent fields. Geographic distance tends to prevent vent‐to‐vent larval transport, but there is no gradual genetic differentiation by depth.

Relative migration analysis showed that the highest migration rate occurs between INF and IZH. Three vent fields (MEK, INF, and IZH), which are geographically close, show bidirectional migration. There is no significant difference between migration from southwest to northeast and from northeast to southwest. In addition, gene flow occurs regardless of depth (no isolation‐by‐depth). Although larvae appear to have buoyancy and vertical swimming ability, active behavior may not be sufficient to migrate long distances (over a few hundred kilometers). This results in limited larval dispersal range (Craddock et al., 1997; Lutz et al., 1984). Population genetic data and the ocean circulation model predict that bidirectional migration patterns are maintained by deeper currents over multiple generations, and the migration per generation mainly occurs between geographically proximal sites. These patterns are complex, due to eddies and countercurrents around the Kuroshio Current (Nakamura et al., 2013). Larvae of gastropods, such as limpets, tend to remain near the bottom or to wander with bottom currents (Mullineaux et al., 2005, 2013). Roterman et al. (2016) reported that *L. concentricus* (formerly *Lepetodrilus* sp.) populations at the East Scotia Ridge (ESR) are significantly differentiated from a population in the South Sandwich Island Arc (SSI), only ~95 km from ESR, but there is a large difference in depth between ESR and SSI (2396–2645 m vs. 1434 m depth). Large depth differences may promote genetic differentiation, or deeper environments may result in adaptive isolation of *Lepetodrilus* populations. Nevertheless, differences in depth are not a barrier to genetic connectivity of *L. nux* in the Okinawa Trough. Megaplumes associated with frequent seafloor volcanic eruptions promote larval transport and such events may maintain genetic connectivity among sites, compensating for differences in depth (Matabos et al., 2008). Therefore, some larvae may be transported by upper currents; however, successful dispersal must be rare. As a counteracting effect, PLD becomes shorter and limits dispersal distance closer to the surface than at greater depth due to accelerated larval metabolism caused by increasing water temperature, compensating for increasing current speed (Mitarai et al., 2016). These new insights may be helpful to establish conservation strategies for hydrothermal vent gastropods against drilling for sea floor resources (Nakamura et al., 2018).

We conclude that connectivity of *L. nux* in the Okinawa Trough is limited by geographic distance (within ~545 km), independent of differences in depth. Previous studies have also reported limited genetic connectivity and differentiation of lecithotrophic vent gastropods. Restricted contemporary gene flow was found in *Ifremeria nautilei* between the Manus Basin and the Fiji–Lau area (Kojima et al., 2000; Thaler et al., 2011; Tran Lu et al., 2022). Moreover, there is no definite genetic break between Fiji and Lau and due to unknown processes, genetic connectivity does not reflect geography (Thaler et al., 2011). Plouviez et al. (2019) further reported that *Lepetodrilus* aff. *schrolli* showed no genetic structure within basins, using 42 nuclear DNA markers, but that it showed large genetic differences between the Manus and Lau Basins. Vent‐related mussels of the genus *Bathymodiolus*, a planktotrophic larval taxon that may be able to remain in the water column or on the surface for up to 1 year (Arellano et al., 2014; Arellano & Young, 2009), showed high genetic similarity among regions (Breusing et al., 2015). The planktotrophic red blood limpet, *Shinkailepas myojinensis*, shows no genetic differentiation between the Okinawa Trough and the Izu‐Ogasawara Arc (~1350 km) based on an analysis of the mitochondrial COI gene, as its larvae are expected to have an extraordinarily long planktotrophic larval period of more than a year, and surface temperature limits their distribution (Yahagi et al., 2017).

A population genomic analysis of *Gigantidas platifrons*, which is taxonomically close to *Bathymodiolus*, also showed no significant genetic differentiation among populations in the Okinawa Trough, based on an analysis of genome‐wide SNPs (Xu et al., 2018). Larvae of *G. platifrons* are planktotrophic, with longer larval duration than lecithotrophic larvae (Xu et al., 2018). Nevertheless, isolation‐by‐distance was detected in vent populations of *G. platifrons* analyzed by genome‐wide SNPs. Low genetic differentiation and bidirectional migration patterns were also shown in *B. nipponica* and *S. crosnieri* in the Okinawa Trough, analyzed by genome‐wide SNPs (Xu et al., 2021, 2024), and there is no isolation‐by‐distance in these two species (Figure S3a). In addition, seep populations contribute to isolation of vent populations due to heterogeneity that disturbs isolation‐by‐distance patterns. *Bathyacmaea nipponica* shows relatively high *F*_ST_ values even in vent populations. *Shinkaia crosnieri* shows lower values of *F*_ST_ in vent populations, and its PLD may be longer than those of other species, such as *B. nipponica*. Its lecithotrophic larvae can disperse throughout vent sites in the Okinawa Trough, despite brooding. Larvae of *G. platifrons* are planktotrophic and have negative buoyancy; however, they disperse mainly in the upper layer, whereas larvae of the other two species are expected to be in the middle and deeper layers (Xu et al., 2024). *Lepetodrilus nux* may have larval behavior and/or population history similar to *G. platifrons*, following patterns of isolation‐by‐distance, though *F*_ST_ values of *L. nux* are higher than those of *G. platifrons* and PLDs of these two species may differ. Planktotrophic larvae can feed in the water column, whereas lecithotrophic larvae depend on egg yolk reserves during the larval period (Vrijenhoek, 2010). This larval trait largely determines larval dispersal capacity and genetic differentiation among sites. Although the genus *Lepetodrilus* is considered lecithotrophic (Vrijenhoek, 2010), larvae of *Lepetodrilus* are potentially planktotrophic, judging from egg size and fecundity (Tyler et al., 2008). Our study of *L. nux* detected genetic differentiation and structure among sites within this region. In addition, *Lepetodrilus*, including *L. nux*, has not been found in the Izu‐Ogasawara Arc, though a related species *Lepetodrilus* aff. *schrolli* is distributed in the Mariana Trough, south of the Izu‐Ogasawara Arc (Johnson et al., 2008). In the ocean circulation model, transport from the Okinawa Trough to Izu‐Ogasawara could occur within 20 days at 500 m (Mitarai et al., 2016). If the planktotrophic larval period is assumed to be several months or more, *L. nux* may be a lecithotrophic larval species. However, larvae of *G. platifrons*, which showed isolation‐by‐distance at vent sites in the Okinawa Trough, is planktotrophic. Further analysis will need to confirm the larval nutrition type of *Lepetodrilus*, though *Pyramidella subglabra*, *G. platifrons*, *B. nipponica*, and *S. crosnieri* inhabit both vents and seeps and their settlement characteristics differ from those of vent‐endemic species, such as *L. nux*. To verify the nutrition type and behavior of *L. nux* larvae, long‐term artificial culture will be helpful to understand larval development and physiological traits, for example, quantitative egg yolk, mortality, buoyancy and swimming behavior, and so on. Furthermore, using high‐resolution genetic markers, more vent‐endemic species, including other planktotrophic limpets, such as *S. myojinensis* in the Okinawa Trough, also need to be analyzed for comparison of genetic differentiation between larval nutrition types.

Ascending to the sea surface effectively shortens larval duration as a result of accelerated metabolism caused by higher water temperatures, especially in lecithotrophic larval species (Figure S2). If *L. nux* larvae are transported via shallow water and survive, larvae may occasionally be able to settle in other hydrothermal vent fields. Such low‐level migration should be sufficient to maintain connectivity among hydrothermal vent fields. Molluscan dispersal period data (O'Connor et al., 2007) are derived from shallow‐water species, and deep‐sea molluscan PLDs of planktotrophic larvae have been estimated (Arellano et al., 2014; Arellano & Young, 2009; Yahagi et al., 2017). *Lepetodrilus nux* is considered lecithotrophic; therefore, predicted PLDs may not be applicable to connectivity or differentiation among hydrothermal vent fields. Actual dispersal range must vary by location because of differences in depth, currents, temperature, and geography. Nevertheless, there is no significant differentiation between the IZH and IRK fields, despite geographic separation of >300 km, and DAPC genetic clusters are very similar. Active hydrothermal vent fields (Gondou) discovered near Kume Island in 2014 (Minami & Ohara, 2017), located between IZH and IRK, may offer genetic connectivity in the Okinawa Trough. Further study of population genetics with more populations may enable us to better understand connectivity and migration patterns of this species.

The ocean circulation model by ROMS estimated that successful migration between hydrothermal vents tends to occur at depths of 700–1000 m. Surface currents may strongly disperse larvae, reducing the rate of successful recruitment. Ocean circulation at greater depths may contribute to short‐distance dispersal to neighboring vent fields at this geographic scale. In one generation, the probability of larval transport is indeed higher at shallower depths than at 1000 m over longer distances, for example, over 400 km, whereas in two generations, it becomes greater at 700 and 1000 m depths, than in shallower water at all geographic distances (Figure 4).

Isolation‐by‐depth is non‐significant; therefore, stochastic recruitment occurs from ~700 m (MEK) to ~1650 m (IRK) depth. However, the Kuroshio Current is very strong along the Okinawa Trough and the ocean circulation model tends to support large potential migration from southwest to northeast. Contrary to expectation, migration from northeast to southwest appears to occur in adjacent areas. Ocean circulation via the Kuroshio Current from southwest to northeast appears likely as a driving force of long‐distance dispersal. Genetic migration rates show bidirectional migration without significant directionality. Discrepancies have been reported between genetic approaches and the ocean circulation model (Breusing et al., 2023; Moody et al., 2019). The timescale difference between genetic approaches via multiple generations with evolutionary history and the ocean circulation model may create such discrepancies. Temporal variation in larval supply potentially has important consequences for population genetic structure (Mullineaux et al., 2005). Historic colonization and larval physiology are crucial for genetic connectivity and these factors vary even among related species (Breusing et al., 2023). However, the *L. nux* PLD is unknown and should be determined to better define the actual dispersal range of this species. PLD variation may contribute to recruitment at both natal and distant vent fields. Further studies of larval behavior, actual dispersal depth, and larval physiology will allow realistic estimates of larval dispersal and survivorship. In addition, “ghost populations” with unknown effective population sizes derived from unsampled or unknown hydrothermal vent fields may support migration, contributing to genetic connectivity. The existence of *Lepetodrilus* species in other regions in the northwestern Pacific may reveal previously unknown populations. Moreover, investigation of hydrothermal vent‐endemic invertebrates using genotypic big data, such as genome‐wide, single‐nucleotide polymorphisms with quantitative trait loci may clarify the distribution and adaptation of this species/genus in the northwestern Pacific.

## CONFLICT OF INTEREST STATEMENT

The authors declare that they have no conflicts of interest.

## Supporting information


Appendix S1

## Data Availability

Microsatellite genotype data in this study are available at the Dryad Digital Repository: https://doi.org/10.5061/dryad.f4qrfj736.
