# Supplementary material for: Ocean circulation contributes to genetic connectivity of limpet populations at deep‐sea hydrothermal vents in a back‐arc basin
Source: Evol Appl. 2024 Jun 17;17(6):e13727. doi: 10.1111/eva.13727 (PMC11183178; doi:10.1111/eva.13727)
Supplement: Supplementary file 1 — Appendix S1 [file EVA-17-e13727-s001.docx]

**Ocean circulation contributes to genetic connectivity of limpet populations at deep-sea hydrothermal vents in a back-arc basin**

Yuichi Nakajima, Masako Nakamura, Hiromi Kayama Watanabe, Jun-ichiro Ishibashi, Hiroyuki Yamamoto, Satoshi Mitarai

**Supporting Information**

**
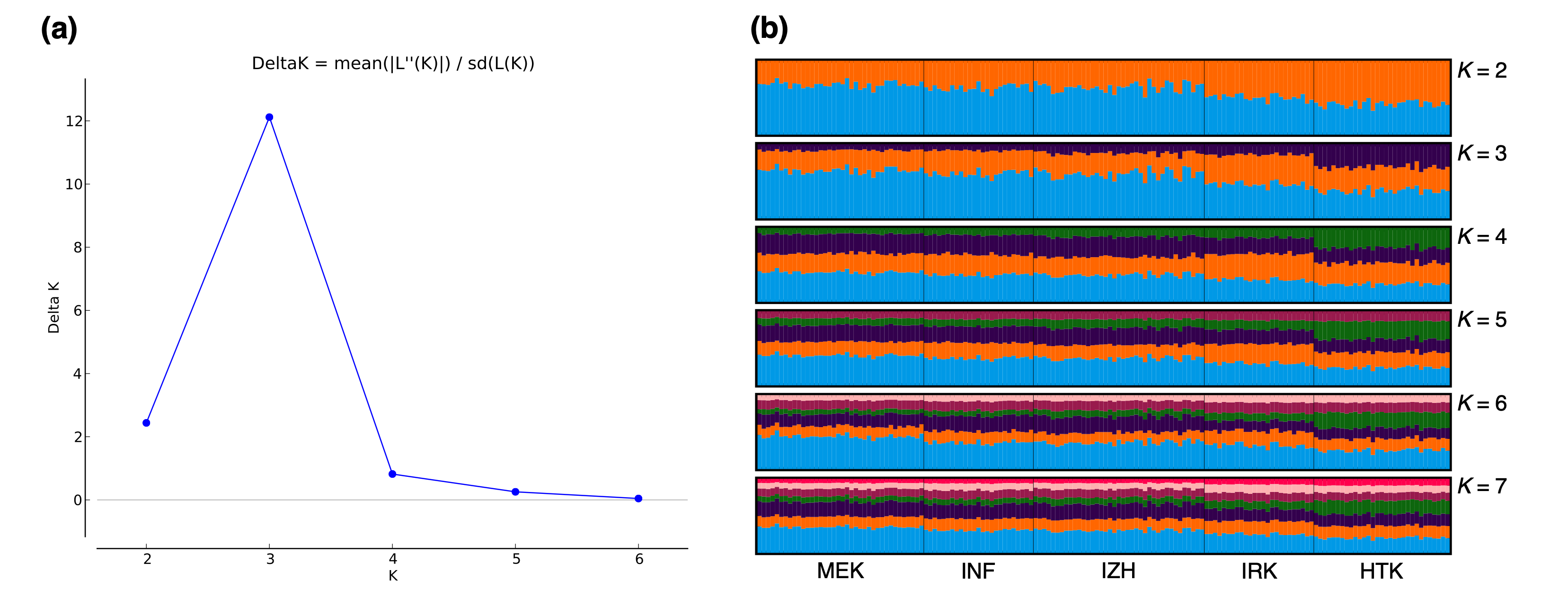
**

**Figure S1.** (a) Δ*K* values (*K* = 2 to *K* = 6) for detecting the number of *K* clusters that best fit the data, suggested by Evanno et al. (2005). (b) STRUCTURE bar plots each *K* (*K* = 2 to *K* = 7).

**
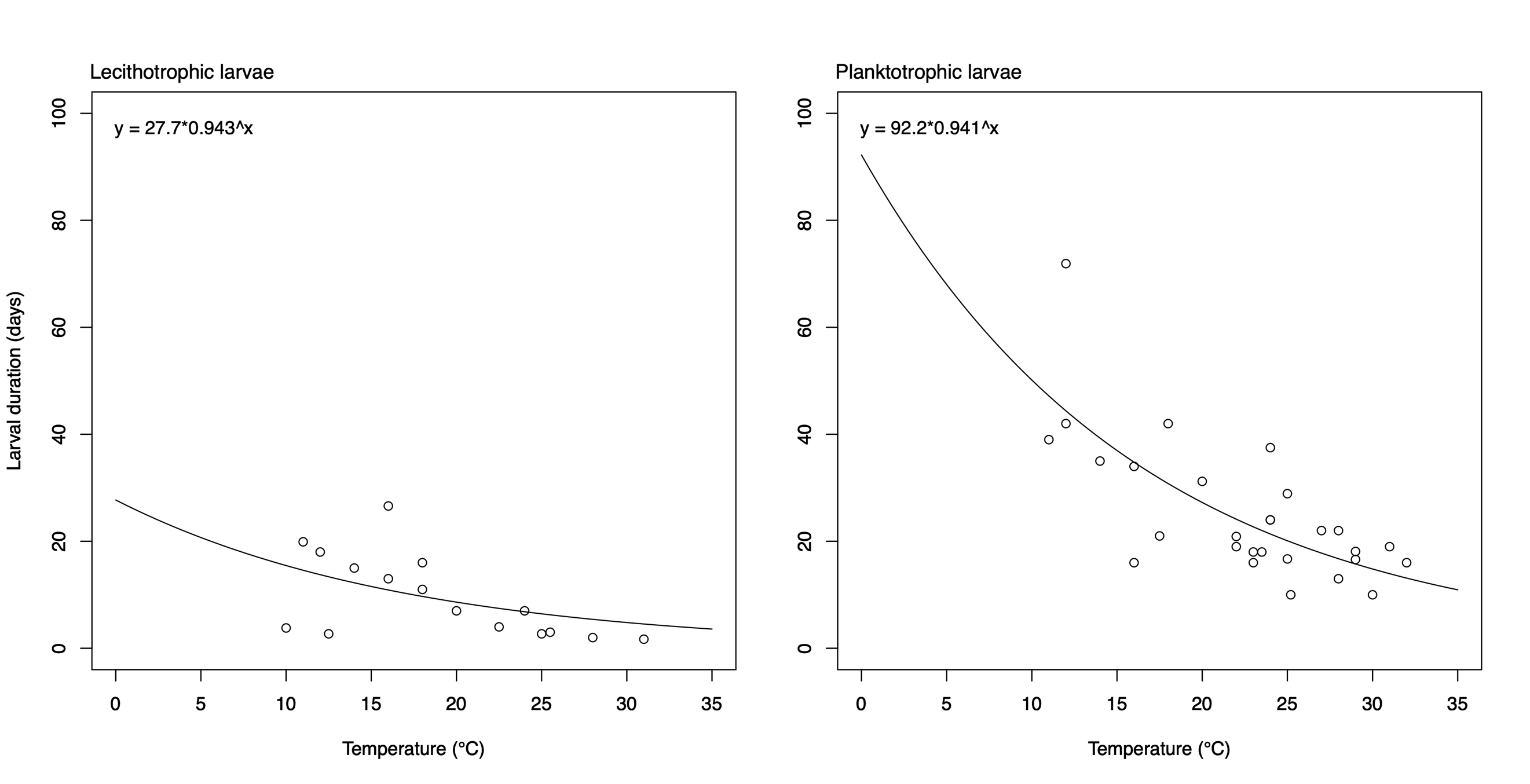
**

**Figure S2.** The relationship between water temperature and larval duration for mollusk species of planktotrophic and lecithotrophic larval types (O’Connor et al., 2007 and references). The exponential curve was produced using nls (nonlinear least squares) function in R.


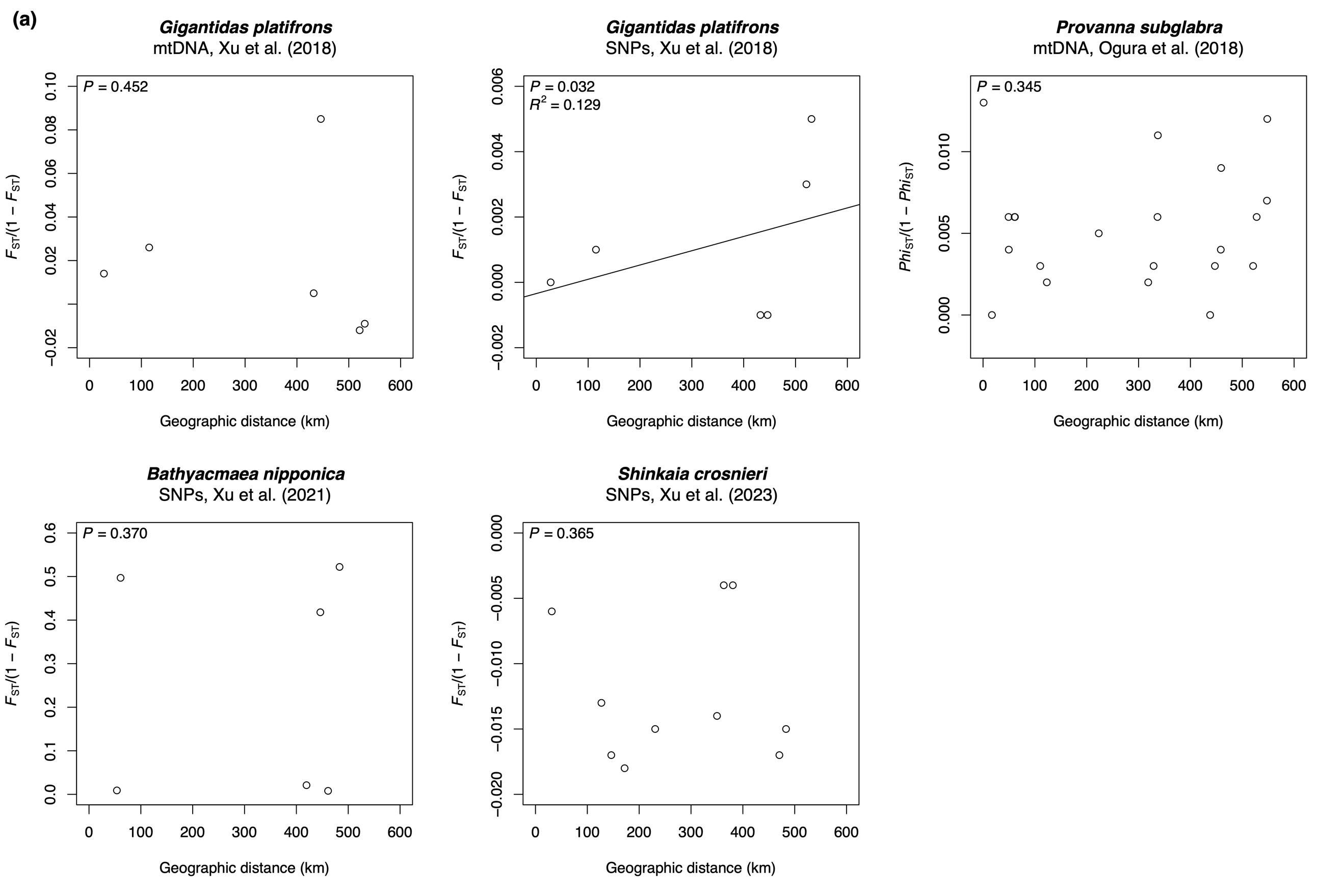


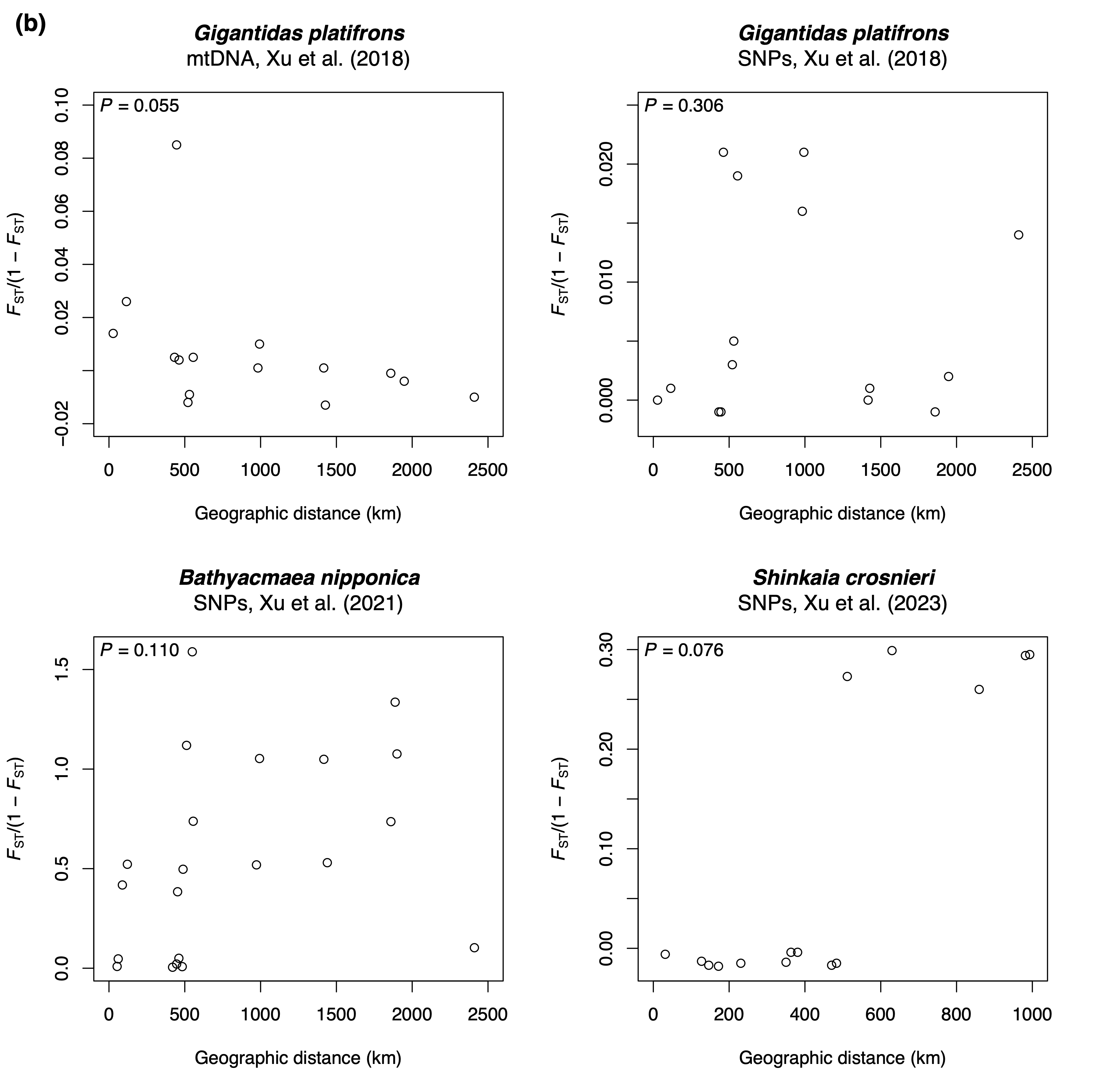


**Figure S3.** (a) Isolation-by-distance among vent sites of five vent species analyzed previously. (b) Isolation-by-distance among all sites (both vent and seep sites) of four vent species except for vent endemic species, *Provanna subglabra*. The genetic differentiation was *F*_ST_ / (1 – *F*_ST_) or *Phi*_ST_ / (1 – *Phi*_ST_) based on the pairwise *F*_ST_ or *Phi*_ST_, respectively. Geographic distance was calculated from coordinates of vent sites.

**Table S1.** Characteristics of 14 polymorphic microsatellite loci developed by Nakajima et al. (2018) and utilized in this study: locus name, primer sequence, repeat motif, size range including U19 sequence, combination of locus and fluorescent labels for multiplex PCR, and GenBank accession number.

| Locus | Repeat motif | Primer sequence (5'-3') | Size range (bp) | Combination of multiplex PCR (fluorescent) | Accession No. |
| --- | --- | --- | --- | --- | --- |
| Lnux_16 | (TGTCG)_12_ | F: ATCATTATCGGTGAAATTCG | 151–207 | A (PET) | AB971597 |
|  |  | R: SP6-TGCATAGAACGTTTGG |  |  |  |
| Lnux_18 | (TCAT)_13_ | F: TGGAGAGTCGCTGAACTGG | 155–195 | B (NED) | AB971598 |
|  |  | R: T7-CCTTTGATACAGAGAACG |  |  |  |
| Lnux_30 | (TAC)_15_(AAT)_4_ | F: T7-ACCGTTACACAGGGATGC | 190–258 | C (NED) | AB971604 |
|  |  | R: CCACGATTTTCTTAAAGGC |  |  |  |
| Lnux_42 | (ATA)_15_ | F: CCCTTGTGAAGGTACTTGCG | 150–253 | B (PET) | AB971608 |
|  |  | R: SP6-ACAAGCATGGGAGCATGG |  |  |  |
| Lnux_54 | (TAC)_20_TAT(TAA)_6_ | F: ACCAGTAGTTGATCGCAGG | 143–203 | B (FAM) | AB971601 |
|  |  | R: U19-TGTTCAGTCTCCAACGGG |  |  |  |
| Lnux_70 | (TGT)_6_TAT(TGT)_4_TAT(TGA)_21_ | F: M13R-TCTTATCATGATCGCGTCG | 159–217 | C (VIC) | AB971619 |
|  |  | R: CAATGTCGGTGAATAAGTGC |  |  |  |
| Lnux_78 | (ATA)_10_ | F: M13R-CTTTGATGGTGTTGAATCG | 90–117 | A (VIC) | AB971622 |
|  |  | R: TCAATGAAGCTCATGAATGC |  |  |  |
| Lnux_79 | (CTT)_3_N_9_(CAT)_3_CAG(CAT)_10_ | F: CCGCGAGTATCATAATGCC | 159–245 | A (VIC) | AB971623 |
|  |  | R: M13R-ATGTAGAAATTGCGCCCG |  |  |  |
| Lnux_81 | (ATA)_10_ | F: T7-TTAAGGGTTTTGTTGGCG | 152–174 | C (NED) | AB971624 |
|  |  | R: AAAGCTCTTTGGAAGTAGCC |  |  |  |
| Lnux_84 | (TCA)_10_ | F: U19-ACCACTGAGCACCTTCGG | 133–163 | A (FAM) | AB971626 |
|  |  | R: CCTGGAGGAGGGACAAGG |  |  |  |
| Lnux_91 | (ATA)_10_ | F: TTTATCGAATGTTGCACAGC | 133–172 | A (NED) | AB971628 |
|  |  | R: T7-TCTTTGTCTGCGCTTCG |  |  |  |
| Lnux_92 | (AAT)_10_ | F: M13R-CCAAAATGACACCAGCACC | 130–160 | B (VIC) | AB971629 |
|  |  | R: TTTCCACATGGAGTCCAGC |  |  |  |
| Lnux_94 | (TAA)_10_ | F: SP6-TTGGTGAAATGTCATGAGG | 119–140 | A (PET) | AB971630 |
|  |  | R: AGTGGGTGGATATGGCG |  |  |  |
| Lnux_95 | (ATT)_10_ | F: SP6-GGTGAAATATTCCCACTGC | 147–196 | C (PET) | AB971631 |
|  |  | R: CACGGTTAAAACTGAATGGG |  |  |  |

**Table S2.** The number of alleles (*N*_A_), private alleles (*P*_A_), observed (*H*_O_) and expected (*H*_E_) heterozygosities, and the index of deviation from HWE (*F*_IS_) for each locus/site. *Significant deviation from HWE (**P* < 0.05, ***P* < 0.01, ****P* < 0.001).

| Location |  | Lnux_16 | Lnux_18 | Lnux_30 | Lnux_42 | Lnux_54 | Lnux_70 | Lnux_78 | Lnux_79 | Lnux_81 | Lnux_84 | Lnux_91 | Lnux_92 | Lnux_94 | Lnux_95 |
| --- | --- | --- | --- | --- | --- | --- | --- | --- | --- | --- | --- | --- | --- | --- | --- |
| MEK | *N*_A_ | 10 | 9 | 11 | 13 | 9 | 18 | 7 | 17 | 8 | 9 | 11 | 7 | 6 | 13 |
|  | *P*_A_ | 1 | 0 | 0 | 2 | 1 | 3 | 0 | 5 | 1 | 3 | 1 | 0 | 0 | 4 |
|  | *H*_O_ | 0.816 | 0.684 | 0.842 | 0.684 | 0.579 | 0.632 | 0.526 | 0.921 | 0.711 | 0.684 | 0.711 | 0.737 | 0.474 | 0.737 |
|  | *H*_E_ | 0.777 | 0.701 | 0.848 | 0.866 | 0.754 | 0.904 | 0.628 | 0.866 | 0.636 | 0.650 | 0.637 | 0.639 | 0.535 | 0.790 |
|  | *F*_IS_ | −0.049 | 0.024 | 0.007 | 0.210*** | 0.232 | 0.302*** | 0.162*** | −0.064 | −0.118 | −0.053 | −0.115*** | −0.154 | 0.115 | 0.067 |
| INF | *N*_A_ | 8 | 9 | 12 | 13 | 9 | 16 | 7 | 16 | 5 | 7 | 9 | 8 | 5 | 9 |
|  | *P*_A_ | 0 | 0 | 1 | 0 | 0 | 0 | 0 | 1 | 0 | 1 | 0 | 0 | 0 | 0 |
|  | *H*_O_ | 0.800 | 0.840 | 0.920 | 0.840 | 0.920 | 0.960 | 0.520 | 0.880 | 0.480 | 0.520 | 0.680 | 0.600 | 0.720 | 0.840 |
|  | *H*_E_ | 0.778 | 0.762 | 0.838 | 0.862 | 0.793 | 0.915 | 0.670 | 0.891 | 0.557 | 0.510 | 0.766 | 0.570 | 0.550 | 0.833 |
|  | *F*_IS_ | −0.029* | −0.102 | −0.097 | 0.026 | −0.160 | −0.049 | 0.223*** | 0.013 | 0.138* | −0.019 | 0.112*** | −0.053 | −0.308 | −0.009 |
| IZH | *N*_A_ | 10 | 8 | 10 | 11 | 14 | 16 | 8 | 15 | 8 | 8 | 8 | 10 | 6 | 9 |
|  | *P*_A_ | 3 | 1 | 0 | 0 | 4 | 0 | 0 | 1 | 1 | 0 | 0 | 0 | 0 | 0 |
|  | *H*_O_ | 0.692 | 0.641 | 0.872 | 0.667 | 0.821 | 0.744 | 0.385 | 0.846 | 0.590 | 0.590 | 0.718 | 0.718 | 0.410 | 0.744 |
|  | *H*_E_ | 0.795 | 0.734 | 0.823 | 0.838 | 0.840 | 0.900 | 0.677 | 0.893 | 0.664 | 0.606 | 0.768 | 0.715 | 0.547 | 0.833 |
|  | *F*_IS_ | 0.129* | 0.127 | −0.060 | 0.204 | 0.023 | 0.174 | 0.431*** | 0.053 | 0.112* | 0.027 | 0.065*** | −0.004 | 0.250 | 0.107 |
| IRK | *N*_A_ | 10 | 10 | 12 | 13 | 9 | 15 | 8 | 14 | 7 | 7 | 8 | 9 | 7 | 10 |
|  | *P*_A_ | 3 | 0 | 1 | 2 | 1 | 2 | 0 | 1 | 0 | 0 | 1 | 1 | 1 | 0 |
|  | *H*_O_ | 0.800 | 0.800 | 0.880 | 0.920 | 0.600 | 0.480 | 0.720 | 0.880 | 0.640 | 0.600 | 1.000 | 0.720 | 0.440 | 0.720 |
|  | *H*_E_ | 0.817 | 0.778 | 0.835 | 0.858 | 0.798 | 0.907 | 0.679 | 0.872 | 0.628 | 0.631 | 0.817 | 0.832 | 0.534 | 0.817 |
|  | *F*_IS_ | 0.021 | −0.028 | −0.054* | −0.072 | 0.248 | 0.471*** | −0.060 | −0.009 | −0.019*** | 0.049* | −0.224 | 0.135 | 0.175 | 0.119*** |
| HTK | *N*_A_ | 10 | 9 | 12 | 13 | 9 | 15 | 9 | 12 | 9 | 8 | 9 | 9 | 6 | 10 |
|  | *P*_A_ | 5 | 0 | 1 | 3 | 1 | 0 | 0 | 0 | 0 | 1 | 1 | 0 | 0 | 1 |
|  | *H*_O_ | 0.839 | 0.774 | 0.806 | 0.742 | 0.935 | 0.613 | 0.839 | 0.806 | 0.613 | 0.774 | 0.774 | 0.774 | 0.419 | 0.774 |
|  | *H*_E_ | 0.816 | 0.672 | 0.833 | 0.866 | 0.822 | 0.907 | 0.754 | 0.887 | 0.666 | 0.748 | 0.804 | 0.839 | 0.492 | 0.784 |
|  | *F*_IS_ | −0.028 | −0.152 | 0.032 | 0.144** | −0.139 | 0.324 | −0.112 | 0.091 | 0.080*** | −0.035 | 0.038 | 0.077 | 0.148 | 0.012 |

**Table S3.** (a) Analysis of molecular variance (AMOVA). Degrees of freedom (d.f.), sum of squares (SS), variance components (Var.), and percentage of variances (%). (b, c) Pairwise *F*_ST_ and *G*″_ST_ values (lower diagonal) and *P*-values (above diagonal) among vent fields. Bold *P*-values show significant differentiation (*P* < 0.05).

| (**a**) AMOVA | d.f. | S.S. | Est. Var. | % |  |
| --- | --- | --- | --- | --- | --- |
| Among sites | 4 | 33.177 | 0.047 | 0.86 |  |
| Within sites | 311 | 1668.500 | 5.365 | 99.14 |  |
| Total | 315 | 1701.677 | 5.412 | 100 |  |
|  |  |  |  |  |  |
| (**b**) pairwise *F*_ST_ | MEK | INF | IZH | IRK | HTK |
| MEK |  | 0.148 | 0.114 | **0.012** | **0.001** |
| INF | 0.003 |  | 0.468 | **0.006** | **0.001** |
| IZH | 0.003 | −0.003 |  | 0.224 | **0.001** |
| IRK | 0.008 | 0.011 | 0.002 |  | 0.065 |
| HTK | 0.023 | 0.023 | 0.012 | 0.005 |  |
|  |  |  |  |  |  |
| (**c**) pairwise *G″*_ST_ | MEK | INF | IZH | IRK | HTK |
| MEK |  | 0.164 | 0.219 | **0.019** | **0.001** |
| INF | 0.011 |  | 0.891 | **0.004** | **0.001** |
| IZH | 0.007 | −0.016 |  | 0.460 | **0.001** |
| IRK | 0.030 | 0.043 | 0.001 |  | 0.109 |
| HTK | 0.092 | 0.098 | 0.050 | 0.019 |  |
